# Serum metabolic signatures associated with maximal voluntary ventilation in native high-altitude Tibetans

**DOI:** 10.1007/s11306-026-02517-2

**Published:** 2026-08-22

**Authors:** Tao Zhou, Jiawei Yang, Qiong Zhang, Haichen Zhang, Lening Chen, Shusheng Luo, Qianqian Xiao, Qinghe Meng, Jianjun Jiang, Labasangzhu Labasangzhu, Dunyou Dunyou, Danbalangjie Danbalangjie, Weidong Hao, Xuetao Wei

**Affiliations:** 1 Department of Toxicology, School of Public Health, Peking University, Beijing, 100191 PR China; 2 Beijing Key Laboratory of Toxicological Research, Risk Assessment for Food Safety, Beijing, 100191 PR China; 3 Department of Maternal and Child Health, School of Public Health, Peking University, Beijing, 100191 PR China; 4 Department of Preventive Medicine, Tibet University Medical College, Lhasa, Tibet, PR China; 5 Key Laboratory of Public Health Safety &Policy Research of Lhasa city, Tibet University, Tibet, PR China; 6 High Altitude Medical Research Center of Tibet University, Lhasa, Tibet, PR China; 7 Nursing Department, Center Hospital of Pulan County, Tibet, Ali Prefecture PR China; 8 People’s Hospital of Ritu County, Tibet, Ali Prefecture PR China

**Keywords:** Native high‑altitude Tibetans, Maximal voluntary ventilation, Serum metabolomics, Elastic net, Multi‑stage screening, Hypoxia‑adapted pulmonary function

## Abstract

**Purpose:**

This study aimed to identify serum metabolites independently associated with maximal voluntary ventilation (MVV) among native high‑altitude Tibetan adults, and explore metabolic correlates of pulmonary ventilatory function under chronic hypoxic exposure.

**Methods:**

Untargeted serum metabolomic profiling comprising 5310 initially detected metabolic features, with 3381 high-quality annotated metabolites retained after strict quality filtering and comprehensive clinical data were collected from 168 native high‑altitude Tibetan adults with complete clinical, phenotypic, and metabolomic data (no missing values in the final analytical dataset). Continuous phenotypic and metabolomic variables were Z‑score‑standardized before analysis. Participants were stratified by MVV quartiles and randomly split into a training set (70%, *n* = 119) and an independent validation set (30%, *n* = 49). A multi‑tier metabolite‑screening pipeline was implemented solely within the training set: tiered‑threshold univariate regression generated candidate metabolites, followed by elastic‑net regularized regression (α = 0.5, lambda.1se). A prespecified fallback rule selected the top‑five metabolites with the smallest univariate P‑values when elastic‑net produced no non‑zero‑coefficient features. Selected candidates were entered into confounder‑adjusted multivariable linear regression, retaining metabolites with *P* < 0.05. Bootstrap resampling assessed screening stability. Ten‑fold cross‑validation evaluated internal performance, and the final model was tested on the independent validation set. Sensitivity analysis was performed by excluding the exogenous drug‑related metabolite to evaluate robustness of core associations.

**Results:**

Three serum metabolites showed independent associations with MVV: N‑Undecanoylglycine (β = −5.237, *P* = 0.002), Isosorbide Dinitrate (β = 0.245, *P* = 0.007), and Hypoglycin B (β = 4.655, *P* = 0.006). The metabolite‑based model yielded a 10‑fold cross‑validated R^2^ of 0.191 in the training set, with predictive R^2^ decreasing to 0.090 in the independent validation set. Bootstrap inclusion frequencies for target metabolites were moderate, suggesting relatively weak marginal metabolite‑MVV effects. Sensitivity analysis confirmed that core endogenous associations were not driven by the exogenous Isosorbide Dinitrate. Formal pathway enrichment was not conducted due to the small number of significant metabolites.

**Conclusions:**

This study identified three serum metabolites independently correlated with MVV in native high‑altitude Tibetans using a multi‑stage metabolome‑wide screening workflow. These metabolites represent preliminary metabolic correlates of ventilatory function under chronic high‑altitude hypoxia. The marked attenuation of predictive performance from internal cross‑validation to external validation reflects limited generalisability of metabolic signatures in moderate‑sized population cohorts. This work emphasises the value of prespecified multi‑tier screening, stability evaluation and fallback strategies for metabolome‑wide association analyses.

**Supplementary Information:**

The online version contains supplementary material available at https://doi.org/10.1007/s11306-026-02517-2.

## Introduction

Monitoring and preserving pulmonary function represents a critical public health priority, especially for native high-altitude Tibetan populations who undergo lifelong chronic hypoxic stress that profoundly reshapes respiratory physiological adaptation (Moore et al. 1998, Beall 2007, West 2012). Although environmental and genetic determinants of lung function in high-altitude residents have been increasingly documented (Simonson et al. 2010, Bigham et al. 2013), the systemic metabolic mechanisms underlying individual differences in ventilatory capacity remain poorly characterized. Given the unique evolutionary and physiological adaptive features of native Tibetans, identifying metabolic signatures linked to pulmonary function may advance our understanding of hypoxia-related respiratory regulation and provide candidate biomarkers for evaluating ventilatory adaptation status (Bigham et al. 2010).

Untargeted metabolomics enables high-throughput profiling of circulating small-molecule metabolites and has become a powerful tool for uncovering molecular correlates of complex physiological phenotypes (Minno et al. 2021). Nevertheless, metabolome-wide association studies (MWAS) encounter common analytical challenges, including ultra-high dimensionality, residual clinical confounding, and unstable variable selection (Bictash et al. 2010). Notably, complex physiological traits such as maximal voluntary ventilation (MVV) are typically regulated by weak and dispersed metabolic signals, which frequently results in null outputs from conventional regularization screening. Traditional single-step screening strategies are therefore prone to false-positive associations or complete feature loss, highlighting the necessity of standardized multi-stage screening frameworks, predefined fallback rules, and rigorous stability validation in metabolomic analyses.

Regularized regression approaches, particularly elastic net regression, effectively handle high-dimensional metabolomic data by balancing variable selection and multicollinearity tolerance (Hong et al. 2023). Compared with single-penalty regularization methods, elastic net (α = 0.5) provides more stable feature retention for complex metabolomic datasets (Friedman et al. 2010). Furthermore, bootstrap resampling and independent dataset validation have been widely recommended to evaluate screening robustness and model generalizability, which substantially improves the reliability of MWAS findings (Zucchini 2000). Despite these methodological advances, metabolomic signatures specifically associated with MVV, a key indicator of overall ventilatory reserve, have not been systematically explored in native high-altitude Tibetans.

To fill this research gap, the present study conducted a comprehensive serum metabolome-wide analysis in 168 native high-altitude Tibetan adults. We adopted a rigorous multi-stage analytical framework integrating tiered univariate filtering, elastic net regularization, and a predefined fallback selection strategy to address potential feature loss during regularized screening. Bootstrap stability evaluation, ten-fold internal cross-validation, and independent external validation were sequentially performed to quantify screening repeatability and model performance after full adjustment for clinical collinearity and confounding factors. This study aimed to identify serum metabolites independently correlated with MVV and characterize potential metabolic correlates of pulmonary ventilatory function under chronic high-altitude hypoxia.

## Materials and methods

### Study population and sample screening

A total of 202 native high-altitude Tibetan adults were initially enrolled in the present study. Strict sample quality control and exclusion criteria were applied to guarantee data reliability. Participants with missing maximal voluntary ventilation (MVV) data (*n* = 8), a documented history of pulmonary diseases (*n* = 12), or incomplete core clinical covariates (*n* = 14) were excluded. After systematic screening, 168 participants with fully complete clinical, phenotypic, and metabolomic data were included in the final analytical cohort, with no missing values observed in any variable. This investigation strictly adhered to the principles of the Declaration of Helsinki, fully safeguarding the life, health, dignity, and autonomy of all participants. Informed consent was obtained from each subject prior to their inclusion in the research. This study was approved by the Medical Ethics Review Committee of Tibet University (2023SQ001).

### Clinical covariate selection and standardized integration

Based on well-established confounding factors affecting pulmonary function in high-altitude populations, a total of 20 preliminary clinical covariates covering six domains were recruited for confounder adjustment, including demographics, anthropometric parameters, socioeconomic status, lifestyle factors, sleep quality, and medical history. The full covariate list comprised: (1) demographics: age, integrated gender-menopause status; (2) anthropometric measurements: height, weight, body mass index (BMI), systolic blood pressure (SBP), diastolic blood pressure (DBP); socioeconomic indicator: per capita monthly income; (4) lifestyle factors: smoking status, cumulative smoking pack-years, drinking status, and metabolic equivalent of task; (5) sleep-related indicators: Breathing difficulty, Sleep latency or Sleep maintenance, Cough snoring, Subjective sleep quality, and Sleep medication; (6) chronic disease history: hypertension and dyslipidemia.

To reduce multicollinearity, eliminate data redundancy, and improve model interpretability, several covariates were rationally integrated and standardized according to physiological logic and epidemiological conventions. First, gender and menopausal status were combined into a unified reproductive characteristic variable to reflect sex-specific physiological differences in ventilatory function. Second, duplicate SBP and DBP measurements were averaged to obtain stable blood pressure values. Third, cumulative smoking pack-years were calculated from daily cigarette consumption and smoking duration to quantify long-term smoking exposure. Fourth, MET values were integrated from the frequency and duration of weekly light, moderate, and heavy physical activities to generate a continuous quantitative index of physical activity level.

### Blood collection and serum metabolite profiling

1. Blood Collection and Serum Preparation

Fasting venous blood was collected from all participants using vacuum blood collection tubes. The collected blood was allowed to clot naturally at room temperature (~ 25 °C) for 30 min, followed by centrifugation at 4 °C and 3,000 rpm for 15 min. The supernatant (serum) was carefully aspirated, aliquoted into sterile centrifuge tubes, and stored at − 80 °C until extraction.

(2) Metabolite Extraction

Serum samples were thawed on an ice-water mixture. For each sample, 90 µL of serum was transferred to a 1.5 mL Eppendorf tube, and 360 µL of pre-chilled protein precipitant (methanol: acetonitrile = 2:1, v/v) containing a mixed internal standard (4 µg/mL) was added. Four stable isotope-labeled internal standards with confirmed vendor information and high purity were used for analytical quality control: L-2-chlorophenylalanine (C2001, Hengchuang Bio, Shanghai, 98.0% purity), succinic acid-d4 (293075-1G, Sigma, 98.0% purity), L-valine-d8 (HY-11124, Haoyuan Bio, Shanghai, 98.0% purity), and cholic acid-D4 (S22155-50 mg, Yuanye Bio, Shanghai, 98.0% purity) The mixture was vortexed vigorously for 1 min, ultrasonicated in an ice-water bath for 10 min, and incubated at − 40 °C overnight to ensure complete protein precipitation. Following incubation, samples were centrifuged at 12,000 rpm for 10 min at 4 °C. A 150 µL aliquot of the supernatant was filtered through a 0.22 μm organic-phase syringe filter, transferred to an LC autosampler vial, and stored at − 80 °C until LC-MS analysis. All samples were extracted on the same day to minimize batch effects. A quality control (QC) sample was prepared by pooling equal volumes of each extracted sample. A pooled quality control (QC) sample was prepared by mixing equal-volume supernatants extracted from all individual study samples to monitor analytical stability throughout the run.

(3) Liquid Chromatography–Mass Spectrometry (LC-MS) Conditions

Untargeted metabolomic profiling was performed using a Waters ACQUITY UPLC I-Class Plus ultra-high-performance liquid chromatography system coupled to a Thermo Q Exactive HF high-resolution mass spectrometer (UHPLC-HRMS). Chromatographic separation was achieved on an ACQUITY UPLC HSS T3 column (100 mm × 2.1 mm, 1.8 μm; Waters Corporation) maintained at 45 °C. The mobile phase consisted of (A) water with 0.1% formic acid and (B) acetonitrile, delivered at a fixed flow rate of 0.35 mL/min. The fixed injection volume was 3 µL. The injection volume was 3 µL. The gradient elution program was as follows: 0–2 min, 5% B; 2–4 min, 5%–30% B; 4–8 min, 30%–50% B; 8–10 min, 50%–80% B; 10–14 min, 80%–100% B; 14–15 min, 100% B; 15.0–15.1 min, 100%–5% B; 15.1–16 min, 5% B.

Mass spectrometric detection was performed in both positive and negative electrospray ionization (ESI) modes to maximize metabolite coverage. The key MS parameters were as follows: spray voltage, + 3,800 V (positive mode)/−3,000 V (negative mode); capillary temperature, 320 °C; auxiliary gas heater temperature, 350 °C; sheath gas flow rate, 35 arbitrary units (Arb); auxiliary gas flow rate, 8 Arb; S-lens RF level, 50. Full-scan data were acquired over the mass range m/z 70–1,050 at a resolution of 60,000 (full width at half maximum). Data-dependent acquisition (DDA) was employed for MS/MS fragmentation at a resolution of 15,000, with normalized collision energy (NCE) set to 10, 20, and 40 (stepped NCE).

(4) Quality Assurance and Quality Control (QA/QC)

A pooled QC sample was injected at the beginning and end of each analytical batch, and additionally after every 15 study samples, yielding a total of 20 QC injections across the run. The QC samples were used to monitor instrument stability, assess data reproducibility, and perform signal correction during data processing. Instrument stability was evaluated by calculating the Pearson correlation between QC samples; higher correlation coefficients indicated greater analytical stability. Prior to each analytical run, the mass spectrometer was calibrated using sodium formate to ensure mass accuracy. Features with a coefficient of variation (CV) > 30% across repeated QC injections were excluded to filter low-reproducibility metabolic features; this 30% CV threshold is a well-accepted standard for untargeted serum metabolomics. All internal standards were consistently detected with low CV values (5%–15%) across QC replicates, confirming reliable instrument stability. No gradient-concentration spiked QC samples were prepared, consistent with standard untargeted metabolomics discovery workflows.

(5) Data Processing and Metabolite Identification

Raw LC-MS data were processed using Progenesis QI version 3.0 (Nonlinear Dynamics, Newcastle, UK). The complete standardized data processing pipeline was strictly implemented as follows: baseline filtering, automatic peak detection, peak integration, software-default retention time alignment, peak grouping, and total ion current (TIC) normalization. For feature filtering, ion features with > 50% missing values across all samples were removed; this lenient threshold was adopted to retain low-abundance trace metabolites, dietary metabolites, and xenobiotics common in serum samples, while eliminating severely undetectable features. Remaining missing values were imputed using the half-minimum (Min/2) value of each feature, a standard method for signals below the limit of detection.

Metabolite identification was performed by matching accurate mass and MS/MS fragmentation spectra against four public and in-house databases: the Human Metabolome Database (HMDB), Lipidmaps, LuMet-Animal (an OE-specific animal metabolome database), and METLIN. A tiered mass tolerance strategy was applied to balance annotation confidence and coverage: precursor mass tolerance of ± 5 ppm and product ion tolerance of ± 10 ppm for rigorously curated HMDB and Lipidmaps databases to reduce false positives; precursor mass tolerance of ± 10 ppm and product ion tolerance of ± 20 ppm for LuMet-Animal and METLIN databases to maximize metabolome coverage.

The Progenesis QI scoring algorithm assigned a composite identification score (maximum 80 points) integrating four orthogonal dimensions: isotopic distribution matching (20 points), fragment ion matching (20 points), retention time consistency (20 points), and adduct ion plausibility (20 points). A composite score cutoff of ≥ 36 (45% of the total maximum score) was applied to retain reliably annotated features, ensuring sufficient matching evidence from at least two or three orthogonal identification dimensions.

A custom four-tier metabolite annotation confidence system (renamed Confidence Tier 1–4 to avoid confusion with community-standard MSI Level classification) was defined explicitly: Tier 1, retention time (RT) deviation ≤ ± 0.3 min AND Fragmentation Score ≥ 45; Tier 2, RT deviation ≤ ± 0.3 min AND 0 ≤ Fragmentation Score < 45; Tier 3, Fragmentation Score ≥ 45 without valid RT matching; Tier 4, Fragmentation Score < 45 without valid RT matching. Notably, this tier labeling only appears in supplementary annotation tables and is not mentioned in the main manuscript text. For key metabolites of interest, manual inspection of matching information was performed to further verify identification confidence. This untargeted discovery study did not purchase full-spectrum authentic reference standards for all metabolite validation; available reference standards were detected in neat solvent for RT and spectral matching rather than biological matrix spiking. A complete list of all identified metabolites with annotation-level information (database identifiers, mass error, retention time, identification score, and MS/MS fragment matching) is provided in Supplementary File 4, with fully revised and clearly defined table headers and detailed column explanations in supplementary readme files.

### Data preprocessing and covariate collinearity diagnosis

A total of 5310 metabolic features were initially detected after peak picking. After missing value filtering and QC-CV filtering, 3381 high-quality annotated metabolic features were retained for all subsequent analyses. Prior to regression modeling, outcome (MVV) and all serum metabolite features were Z‑score standardized. Categorical clinical covariates were not standardized; only continuous clinical covariates underwent Z‑score transformation. Confounder adjustment was implemented via residualization: clinical covariates were regressed out from both MVV and individual metabolites, and the resulting residuals were used for subsequent univariate screening. For preliminary covariates, multicollinearity was assessed using the variance inflation factor (VIF). Variables with VIF < 10 were retained. After collinearity filtering, a total of 17 independent clinical covariates were finalized for subsequent model adjustment.

### Stratified dataset partitioning

To avoid data leakage and ensure rigorous model validation, the entire cohort was stratified by MVV quartiles and randomly split into a training set (70%, *n* = 119) and an independent external validation set (30%, *n* = 49). All metabolite screening, variable selection, model training, and internal validation procedures were performed exclusively in the training set. The independent validation set was not involved in any screening or training processes and was only used to evaluate external model generalizability.

### Multi-stage metabolite screening strategy

A multi-layered robust screening framework was constructed on the training set to screen serum metabolites associated with MVV. Prior to metabolite screening, we established a baseline linear regression model with MVV as the dependent variable and all predefined covariates as independent predictors to calculate covariate-adjusted MVV residuals; this baseline covariate-only model explained 22.85% of MVV variation (R^2^ = 0.2285). All categorical covariates were converted into dummy variables before regression fitting for standardized confounding elimination as shown in Table 1. In the first screening stage, univariate linear regression was performed individually for each metabolite against the adjusted MVV residuals, followed by three-tier hierarchical threshold filtering to preliminarily screen candidate metabolites. The R^2^ values of all single-metabolite univariate models ranged from 0.0000 to 0.1113, with a median of 0.0040 and a mean of 0.0081. Since no metabolite reached significance after FDR correction, we adopted successive nominal thresholds of (*P* < 0.05) and (*P* < 0.01) to expand the candidate pool for subsequent regularized regression. In the second stage, elastic net regularized regression (alpha = 0.5), lambda.1se) was adopted for high-dimensional dimensionality reduction and stable feature selection. To prevent null screening outcomes and guarantee analytical robustness, a fallback screening rule was predefined: elastic net modeling was executed when no fewer than two candidate metabolites were available. If all metabolites obtained zero regression coefficients after regularization, the top five metabolites with the smallest univariate *P* values would be retained as alternative candidate features.

**Table 1:** Distribution of the 17 covariates in the training and validation sets

Variable	Training set	Independent validation set
SBP	120.00(106.75, 135.50)	128.00(113.00, 137.50)
DBP	81.00(75.00, 88.75)	87.00(79.50, 91.00)
Age	58.00(41.50, 69.50)	62.00(49.00, 70.00)
Household income		
< 3000 (1)	40(33.6%)	13(26.5%)
3000–4999 (2)	37(31.1%)	16(32.7%)
5000–7999	21(17.6%)	10(20.4%)
8000–9999(4)	8(6.7%)	2(4.1%)
10,000–14,999(5)	10(8.4%)	6(12.2%)
> 15,000(6)	3(2.5%)	2(4.1%)
MET	1070.00 (626.50, 3066.00)	1197.00 (579.00, 2856.00)
Sleep latency		
None(1)	76(63.9%)	29(59.2%)
Less than once a week(2)	18(15.1%)	9(18.4%)
Once or twice a week	12(10.1%)	6(12.2%)
Three or more times a week(4)	13(10.9%)	5(10.2%)
Sleep maintenance		
None(1)	70(58.8%)	26(53.1%)
Less than once a week(2)	28(23.5%)	12(24.5%)
Once or twice a week	9(7.6%)	7(14.3%)
Three or more times a week(4)	12(10.1%)	4(8.2%)
Breathing difficulty		
None(1)	107(89.9%)	39(79.6%)
Less than once a week(2)	5(4.2%)	6(12.2%)
Once or twice a week	4(3.4%)	1(2.0%)
Three or more times a week(4)	3(2.5%)	3(6.1%)
Cough snoring		
None(1)	89(74.8%)	34(69.4%)
Less than once a week(2)	18(15.1%)	6(12.2%)
Once or twice a week	7(5.9%)	7(14.3%)
Three or more times a week(4)	5(4.2%)	2(4.1%)
Subjective sleep quality		
Very good(1)	43(36.1%)	18(36.7%)
Fairly good(2)	63(52.9%)	22(44.9%)
Fairly bad	10(8.4%)	7(14.3%)
Very bad(4)	3(2.5%)	2(4.1%)
Sleep medication		
None(1)	115(96.6%)	46(93.9%)
Less than once a week(2)	2(1.7%)	2(4.1%)
Three or more times a week(4)	2(1.7%)	1(2.0%)
Smoking status		
Never smoker(0)	86(72.3%)	39(79.6%)
Ever smoker(1)	33(27.7%)	10(20.4%)
Pack_years	0.00(0.00, 0.00)	0.00(0.00, 0.00)
Alcohol drinking		
Ever drinker(1)	54(45.4%)	20(40.8%)
Never drinker(2)	65(54.6%)	29(59.2%)
Hypertension		
Yes(1)	28(23.5%)	9(18.4%)
No(2)	91(76.5%)	40(81.6%)
Dyslipidemia		
Yes(1)	13(10.9%)	10(20.4%)
No(2)	106(89.1%)	39(79.6%)
Sex_menopause status		
Male(0)	45(37.8%)	17(34.7%)
Premenopausal female(1)	41(34.5%)	14(28.6%)
Postmenopausal female(2)	33(27.7%)	18(36.7%)
MVV	36.44(29.02, 50.72)	40.03(30.75, 49.19)

### Adjusted multivariable linear regression model

Candidate metabolites obtained from multi‑stage screening were incorporated into multivariable linear regression models, with Z‑score‑standardized MVV as the dependent variable and the 17 filtered clinical confounders fully adjusted. Metabolites with *P* < 0.05 in the fully adjusted model were considered independently associated with MVV. After multivariable adjustment, three serum metabolites were identified as core independent metabolic correlates of pulmonary ventilatory function. A sensitivity analysis was further performed by excluding the exogenous drug‑related metabolite (Isosorbide Dinitrate), repeating the full multi‑stage analytical pipeline to test the robustness of endogenous metabolite‑MVV associations.

### Model validation and stability evaluation

Multiple validation approaches were adopted to comprehensively assess model performance and screening stability. Ten-fold cross-validation was conducted in the training set to evaluate internal predictive performance and calculate the average cross-validated R^2^. Bootstrap resampling analysis was further performed to quantify the stability and repeatability of metabolite screening outcomes. Finally, the optimized metabolite model was tested in the independent validation set to evaluate external generalization capability, and the corresponding predictive R^2^ was determined.

## Result

### Baseline characteristics of the study population

**Fig. 1:**
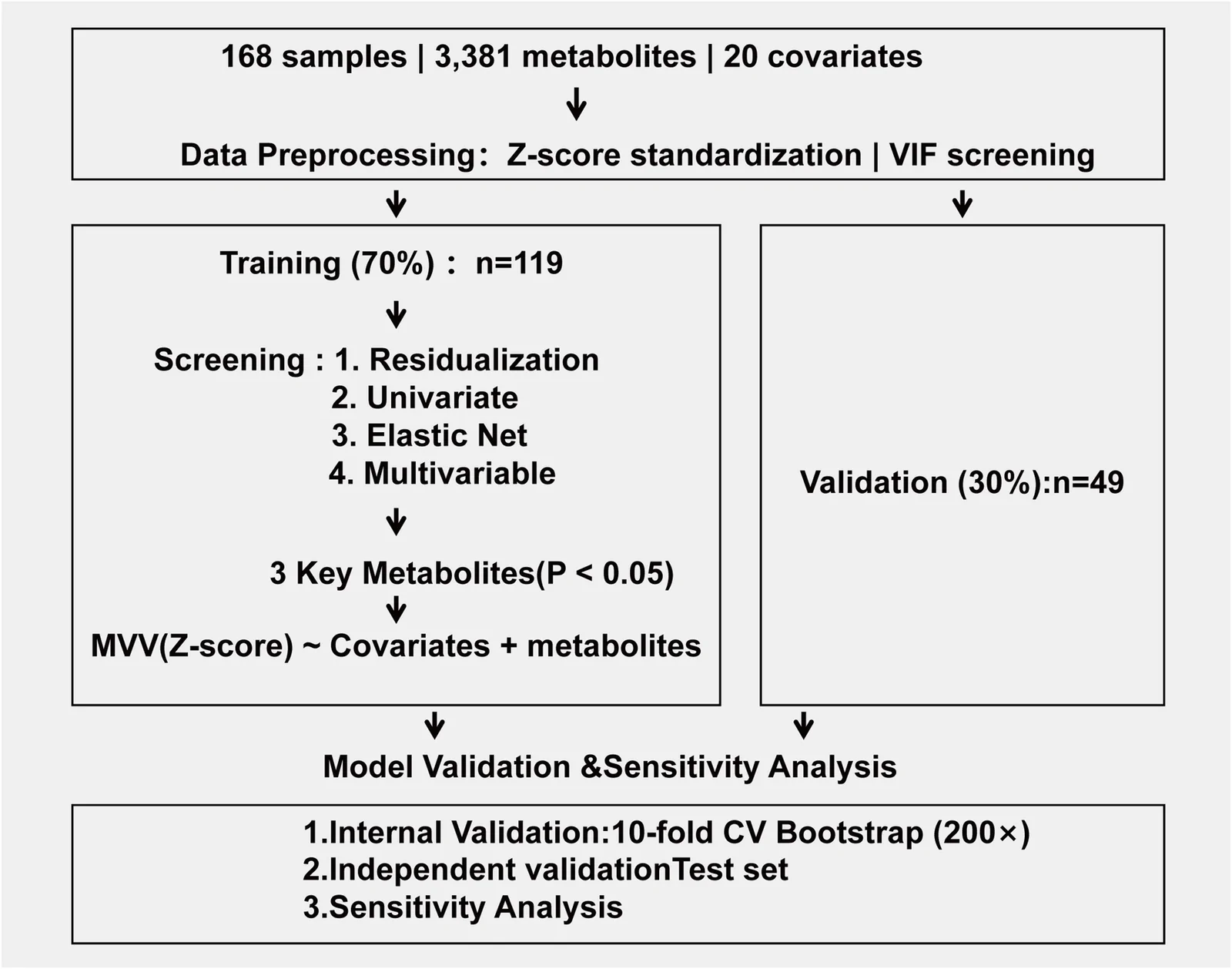
Analytical workflow for identifying MVV-associated metabolites. After preprocessing (Z-score standardization, VIF screening), 168 participants were split into training (*n* = 118) and validation (*n* = 50) sets. A four-step screening pipeline (residualization, univariate regression, Elastic Net, multivariable regression) identified three key metabolites. Model validation included 10-fold CV, bootstrap, sensitivity analysis (exclusion of exogenous metabolites), and independent validation

After rigorous quality control, a total of 168 participants were ultimately enrolled in the present study from an initial pool of 202 high-altitude Tibetan adult samples. Twenty clinical covariates were initially collected for potential confounding adjustment. Prior to statistical modeling, multicollinearity screening was performed with a variance inflation factor (VIF) threshold of 10. Three covariates with severe multicollinearity were excluded, and the remaining 17 independent clinical variables were adopted for all subsequent analyses. All statistical analyses were conducted in R 4.3 with a fixed random seed (12345) to ensure full result reproducibility. The enrolled population was stratified by MVV quartiles and randomly divided into a training set (*n* = 119) and an independent validation set (*n* = 49) at a 7:3 ratio. Baseline characteristics of the two subsets, including demographics, anthropometrics, lifestyle factors, sleep indicators, and medical histories, were summarized in Table 1. Inter-group balance was evaluated using Wilcoxon rank-sum tests, Chi-square tests, or Fisher’s exact tests as appropriate. All covariates exhibited no significant difference between the two groups (all *P* > 0.05), indicating reasonable and unbiased dataset partitioning. Detailed baseline comparison statistics for all original covariates are provided in Supplementary File1. Baseline profiles were presented with raw original values, while Z-score standardization was applied to continuous phenotypic and metabolomic variables before regression analysis. The overall analytical procedure is illustrated in Fig. 1.

### Multi-stage screening of MVV-associated serum metabolites

All metabolite‑screening procedures were strictly restricted to the training dataset. A three‑tier hierarchical screening pipeline integrating univariate regression, tiered‑threshold filtering, elastic‑net regularization (α = 0.5, lambda.1se), and a pre‑specified fallback strategy was implemented to select candidate metabolites. In the first tier, univariate linear regression was performed for each metabolite against MVV residuals adjusted for clinical covariates; no metabolite satisfied the false‑discovery‑rate‑corrected significance threshold of FDR < 0.05. We then relaxed the threshold to nominal univariate *P* < 0.05, yielding 109 candidate metabolites for elastic‑net regularization; however, this model returned no metabolites with non‑zero coefficients. Given that ventilatory capacity (MVV) is shaped by multifactorial physiological regulation and individual metabolite‑MVV marginal associations may be weak, we further expanded the screening margin by adopting a looser univariate threshold of *P* < 0.01, which retained 255 metabolites (Supplementary File 1). Nevertheless, elastic‑net regression still failed to output features with non‑zero coefficients under this enlarged candidate pool. Following our pre‑defined analytical protocol, the top‑five metabolites with the smallest univariate P‑values were therefore selected as candidate features for subsequent multivariable regression.

**Fig. 2:**
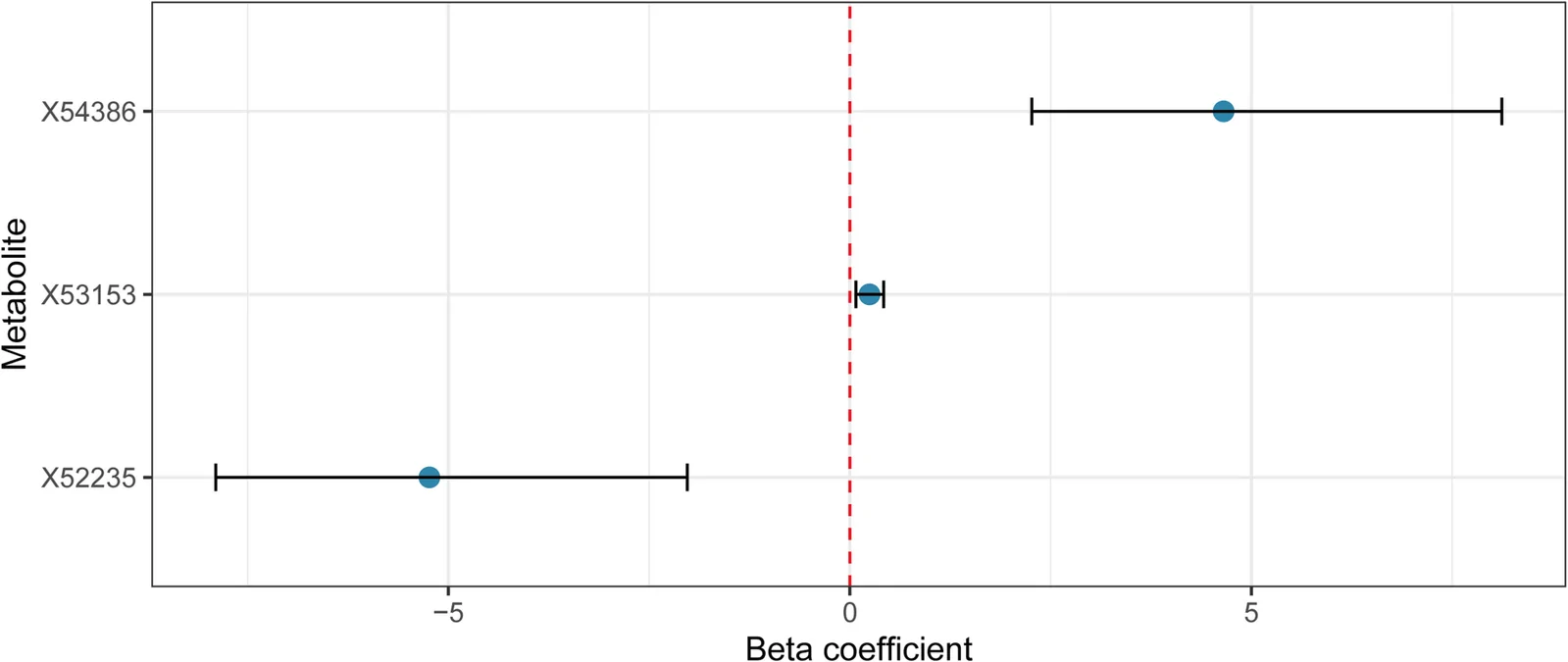
Associations between three identified serum metabolites and maximal voluntary ventilation (MVV). Dot plot illustrating effect sizes (β) and corresponding 95% confidence intervals derived from multivariable linear regression fully adjusted for 17 clinical covariates. The metabolites correspond to X52235 (N‑Undecanoylglycine, β = −5.237, *P* = 0.002), X53153 (Isosorbide Dinitrate, β = 0.245, *P* = 0.007), and X54386 (Hypoglycin B, β = 4.655, *P* = 0.006). Metabolite IDs are shown in the plot, with full metabolite names annotated in the figure legend

After full adjustment for the 17 retained clinical confounders within multivariable linear‑regression models, three serum metabolites were identified as independently and significantly associated with MVV (*P* < 0.05), and these association results are visualized in Fig. 2. Specifically, N‑Undecanoylglycine was negatively associated with MVV (β = −5.237, *P* = 0.002), whereas Isosorbide Dinitrate (β = 0.245, *P* = 0.007) and Hypoglycin B (β = 4.655, *P* = 0.006) exhibited positive associations with MVV.

### Predictive performance, screening stability and sensitivity analysis of the metabolite‑based model

To quantitatively evaluate the predictive performance and robustness of the final multivariable metabolite model, ten‑fold cross‑validation (10‑CV) was performed within the training set, yielding a cross‑validated R^2^ of 0.191. The full model achieved a training R^2^ of 0.424 and an independent validation R^2^ of 0.090, with a slightly lower 10‑CV R^2^ of 0.191. Key predictive metrics for both the primary model and sensitivity‑analysis model are summarised in Table 2. The evident attenuation of predictive accuracy from internal cross‑validation to external independent validation indicated limited generalization ability of the model, which may be attributable to the relatively modest sample size and the complex, multifactorial nature of pulmonary ventilatory variation in high‑altitude native populations. Bootstrap resampling analysis was further applied to assess the stability of metabolite screening. The top‑ten metabolites ranked by bootstrap inclusion frequency are visualized in Supplementary Figure S2, and raw bootstrap outputs are provided in Supplementary File 2. The top three candidate metabolites exhibited moderate bootstrap inclusion frequencies (X53153: 0.205; X54386: 0.125; X52235: 0.110), suggesting that individual metabolite‑MVV marginal associations were relatively weak and susceptible to subtle sampling variation. This moderate stability reflects the inherent complexity of MVV regulation, which is modulated by extensive physiological and environmental factors rather than dominated by a small number of strong metabolic signals.

**Table 2:** Predictive performance of the primary metabolite model and sensitivity analysis model

Model	Training-setR^2^	10-fold CV R^2^	Validation-set R^2^
Primary model (three metabolites)	0.424	0.191	0.090
Sensitivity model (excluding X53153)	0.577	0.169	0.189

Footnote: Comparison of predictive performance between the primary three-metabolite model and the sensitivity model excluding the exogenous metabolite X53153 (Isosorbide Dinitrate). R^2^ values correspond to the training-set coefficient of determination, ten-fold cross-validated R^2^ in the training set, and independent validation-set R^2^, respectively

Considering that one of the three identified metabolites (Isosorbide Dinitrate, X53153) is an exogenous drug‑related metabolite and detailed medication information was unavailable in the present cohort, we performed a sensitivity analysis by excluding this exogenous metabolite and repeating the entire multi‑stage screening and modeling pipeline. Corresponding performance metrics from the sensitivity analysis are also presented in Table 2. Notably, the sensitivity model yielded improved predictive performance, with a training R^2^ of 0.577 and an independent validation R^2^ of 0.189 after excluding the exogenous feature. Detailed regression coefficients and P‑values obtained from the sensitivity‑analysis multivariable model are provided in Supplementary File 3. The retained stable endogenous metabolite associations and improved external predictive capacity demonstrated that the core metabolic signatures associated with MVV were not dependent on exogenous drug interference, further supporting the reliability of the primary findings.

In addition, given that only three independent metabolites were identified, formal pathway enrichment analysis was not conducted, as such a small set of molecular markers cannot provide statistically robust biological pathway inference. Collectively, although the final metabolite‑based model exhibited moderate predictive power, the relatively low explanatory capacity may objectively reflect the genuine weak metabolic susceptibility of ventilatory phenotypic variation in high‑altitude Tibetan populations, rather than insufficient model fitting.

## Discussion

The present study systematically explored serum metabolic signatures associated with maximal voluntary ventilation (MVV) among native high-altitude Tibetans based on a rigorous multi-stage metabolome-wide analytical framework. After standardized sample screening, optimized clinical confounder adjustment, stable feature selection, and dual internal and external model validation, three serum metabolites, including N-Undecanoylglycine, Isosorbide Dinitrate, and Hypoglycin B, were identified as independent correlates of pulmonary ventilatory function in this hypoxic-adapted population. These findings provide preliminary metabolomic insights into individual differences in ventilatory capacity under chronic high-altitude hypoxia, complementing existing genetic and environmental studies on high-altitude pulmonary adaptation.

A core strength of this study lies in its standardized and robust analytical strategy tailored for high-dimensional metabolomic data. Unlike conventional single-step metabolite screening approaches that are prone to false-positive results and poor reproducibility (Broadhurst and Kell 2006), the current study integrated univariate tiered threshold filtering, elastic net regularization, and a predefined fallback screening rule to balance the efficiency and stability of metabolite mining (Chong et al. 2018, Xia et al. 2009). Elastic net regression (α = 0.5) was adopted to overcome the limitations of single-penalty regularization, effectively tolerating metabolite multicollinearity and retaining potentially meaningful weak-effect metabolic features, which is highly suitable for untargeted metabolomic data with ultra-high dimensionality (Chamlal et al. 2024). In addition, this study optimized clinical confounding adjustment based on population characteristics: 20 original clinical variables were rationally integrated, screened, and filtered by collinearity diagnosis to obtain 17 independent covariates, avoiding biased association results caused by redundant or highly collinear clinical factors (Liu et al. 2024). Strict dataset partitioning was also implemented, with all metabolite screening and model training procedures completed exclusively in the training set to eliminate data leakage, while independent external validation was used to objectively evaluate model generalizability (Joeres et al. 2026).

Among the three identified metabolites, N-Undecanoylglycine showed a significant negative correlation with MVV. As a medium-chain fatty acid glycine conjugate, acyl glycines are closely linked to lipid metabolism imbalance and oxidative stress response in respiratory tissues (Isa et al. 2024, Tan et al. 2010, Newgard 2017). Under chronic high-altitude hypoxic stress, abnormal accumulation of N-Undecanoylglycine may reflect disrupted lipid homeostasis in the respiratory system, partially explaining individual declines in ventilatory reserve function (Murray et al. 2018). By contrast, Isosorbide Dinitrate and Hypoglycin B were positively associated with MVV. Notably, Isosorbide Dinitrate is an exogenous drug-derived metabolite rather than an endogenous metabolic molecule, which acts as a classic nitric oxide donor to improve microcirculation and alleviate hypoxic vasoconstriction in lung tissues, potentially contributing to preserved pulmonary ventilation function (Murray et al. 2018, Beall et al. 2012). Hypoglycin B, a characteristic amino acid derivative, may participate in hypoxic metabolic adaptation and energy metabolism regulation in plateau residents, supporting the stability of pulmonary physiological function under long-term hypoxic exposure (D’Alessandro et al. 2016, Chicco et al. 2018). Collectively, these three metabolites participate in multiple biological processes including lipid metabolism, microcirculation regulation, and hypoxic adaptation, suggesting that MVV variation in native high-altitude Tibetans is modulated by complex multi-dimensional metabolic patterns (Lewis et al. 2008).

Notably, the metabolite-based predictive model exhibited a noticeable decrease in predictive performance from internal cross-validation to external validation. The model achieved a moderate predictive R^2^ of 0.191 in ten-fold cross-validation of the training set, whereas the external validation R^2^ dropped to 0.090. This attenuation is a common phenomenon in population-based metabolomic predictive studies and can be attributed to multiple objective limitations (Tzoulaki et al. 2014, Ganna et al. 2014). First, the single-center cohort and moderate sample size (*n* = 168) inevitably limited the statistical power and population representativeness of the study, making the model susceptible to subtle population heterogeneity and random noise during external validation (Button et al. 2013). Second, although comprehensive clinical confounding adjustment was performed based on collected baseline indicators, the current study only recorded histories of hypertension and dyslipidemia without detailed information on individual medication use. This limitation makes it impossible to correct for the potential confounding effect of exogenous drug exposure, which may interfere with the association signal of the drug-derived metabolite Isosorbide Dinitrate (Suhre et al. 2011). Third, due to the limited number of final effective metabolites, formal metabolic pathway enrichment analysis could not be conducted, which restricted the systematic interpretation of the underlying metabolic mechanisms linking metabolites and pulmonary ventilation function (Xia and Wishart 2010).

In addition, several methodological limitations of the current multi-stage screening framework should be acknowledged. The preset fallback strategy of retaining top five metabolites with the smallest univariate P values when elastic net yields no valid features ensures the integrity of analytical workflow, but it may introduce marginal weak-effect metabolites and increase the uncertainty of model fitting (Meinshausen et al. 2010). Meanwhile, bootstrap stability verification confirmed the repeatability of the screening process, but the relatively small validation set sample size weakened the persuasive power of external generalization results (Steyerberg et al. 2010). Furthermore, this study only focused on the cross-sectional correlation between serum metabolites and MVV, and cannot clarify the causal direction of metabolic variation and pulmonary function differences under chronic hypoxia (Lawlor et al. 2008).

## Conclusion

In conclusion, this study identified three serum metabolites independently associated with MVV in native high‑altitude Tibetans through a standardized multi‑stage metabolomic screening and validation strategy. Further sensitivity analysis confirmed that the core metabolic signatures linked to ventilatory function were robust and not dependent on exogenous drug‑derived metabolites, supporting the reliability of the present findings. These results provide preliminary metabolic evidence for individual differences in pulmonary ventilatory adaptation under chronic high‑altitude hypoxia.

The metabolite‑based model presented moderate predictive performance and screening stability, which likely reflects the multifactorial and weakly regulated nature of pulmonary function in plateau populations. Owing to the limited number of candidate metabolites, pathway enrichment analysis was not feasible in this study. Despite inherent limitations including a moderate sample size, single‑center cross‑sectional design, and incomplete medication information, this work offers potential metabolic biomarkers for evaluating high‑altitude respiratory adaptation.

Future large‑scale prospective cohorts and functional experiments are needed to validate the causal relationships between key metabolites and hypoxic pulmonary adaptation and further elucidate the metabolic regulatory mechanisms underlying high‑altitude respiratory physiological phenotypes.

## Supplementary Information

Below is the link to the electronic supplementary material.

Supplementary Material 1

Supplementary Material 2

Supplementary Material 3

Supplementary Material 4

Supplementary Material 5

## Data Availability

No datasets were generated or analysed during the current study.

## Declarations

### AI use statement

Polish the fluency of the article.

## References

[CR1] Beall, C. M. (2007). Two routes to functional adaptation: Tibetan and Andean high-altitude natives. *Proceedings of the National Academy of Sciences of the United States of America*, *104*, 8655–8660. 10.1073/pnas.0701985104 PMC1876443 17494744

[CR2] Beall, C. M., Laskowski, D., & Erzurum, S. C. (2012). Nitric oxide in adaptation to altitude. *Free Radical Biology And Medicine*, *52*, 1123–1134. 10.1016/j.freeradbiomed.2011.12.028 PMC3295887 22300645

[CR3] Bictash, M., Ebbels, T. M., Chan, Q., Loo, R. L., Yap, I. K., Brown, I. J., & Elliott, P. (2010). Opening up the" Black Box": Metabolic phenotyping and metabolome-wide association studies in epidemiology. *Journal of Clinical Epidemiology,**63*(9), 970–979. 10.1016/j.jclinepi.2009.10.001 PMC4048926 20056386

[CR4] Bigham, A. W., Wilson, M. J., Julian, C. G., Kiyamu, M., Vargas, E., Leon-Velarde, F., & Shriver, M. D. (2013). Andean and Tibetan patterns of adaptation to high altitude. *American Journal of Human Biology,**25*(2), 190–197. 10.1002/ajhb.22358 23348729

[CR5] Bigham, A., Bauchet, M., Pinto, D., Mao, X., Akey, J. M., Mei, R., & Shriver, M. D. (2010). Identifying signatures of natural selection in Tibetan and Andean populations using dense genome scan data. *PLoS Genetics,**6*(9), Article e1001116. 10.1371/journal.pgen.1001116 PMC2936536 20838600

[CR6] Broadhurst, D. I., & Kell, D. B. (2006). Statistical strategies for avoiding false discoveries in metabolomics and related experiments. *Metabolomics*, *2*, 171–196. 10.1007/s11306-006-0037-z

[CR7] Button, K. S., Ioannidis, J. P., Mokrysz, C., Nosek, B. A., Flint, J., Robinson, E. S., & Munafò, M. R. (2013). Power failure: Why small sample size undermines the reliability of neuroscience. *Nature Reviews Neuroscience,**14*(5), 365–376. 10.1038/nrn3475 23571845

[CR8] Chamlal, H., Benzmane, A., & Ouaderhman, T. (2024). Elastic net-based high dimensional data selection for regression. *Expert Systems with Applications*, *244*, 122958. 10.1016/j.eswa.2023.122958

[CR9] Chicco, A. J., Le, C. H., Gnaiger, E., Dreyer, H. C., Muyskens, J. B., D’Alessandro, A., & Roach, R. C. (2018). Adaptive remodeling of skeletal muscle energy metabolism in high-altitude hypoxia: Lessons from AltitudeOmics. *Journal of Biological Chemistry,**293*(18), 6659–6671. 10.1074/jbc.RA117.000470 PMC5936810 29540485

[CR10] Chong, J., Soufan, O., Li, C., Caraus, I., Li, S., Bourque, G., & Xia, J. (2018). MetaboAnalyst 40: Towards more transparent and integrative metabolomics analysis. *Nucleic Acids Research,**46*(W1), W486–W494. 10.1093/nar/gky310 PMC6030889 29762782

[CR11] D’Alessandro, A., Nemkov, T., Sun, K., Liu, H., Song, A., Monte, A. A., & Roach, R. C. (2016). AltitudeOmics: Red blood cell metabolic adaptation to high altitude hypoxia. *Journal of Proteome Research,**15*(10), 3883. 10.1021/acs.jproteome.6b00733 PMC5512539 27646145

[CR12] Di Minno, A., Gelzo, M., Stornaiuolo, M., Ruoppolo, M., & Castaldo, G. (2021). The evolving landscape of untargeted metabolomics. *Nutrition Metabolism and Cardiovascular Diseases*, *31*, 1645–1652. 10.1016/j.numecd.2021.01.008 33895079

[CR13] Friedman, J., Hastie, T., & Tibshirani, R. (2010). Regularization Paths for Generalized Linear Models via Coordinate Descent. *Journal Of Statistical Software*, *33*, 1–22. PMC2929880 20808728

[CR14] Ganna, A., Salihovic, S., Sundström, J., Broeckling, C. D., Hedman, Å. K., Magnusson, P. K., & Ingelsson, E. (2014). Large-scale metabolomic profiling identifies novel biomarkers for incident coronary heart disease. *PLoS Genetics,**10*(12), Article e1004801. 10.1371/journal.pgen.1004801 PMC4263376 25502724

[CR15] Hong, F., Tian, L., & Devanarayan, V. (2023). Improving the robustness of variable selection and predictive performance of regularized generalized linear models and cox proportional hazard models. *Mathematics (Basel)*. 10.3390/math11030557 PMC10660556 37990696

[CR16] Isa, A. M., Sun, Y., Wang, Y., Li, Y., Yuan, J., Ni, A., & Chen, J. (2024). Transcriptome analysis of ovarian tissues highlights genes controlling energy homeostasis and oxidative stress as potential drivers of heterosis for egg number and clutch size in crossbred laying hens. *Poultry Science,**103*(1), Article 103163. 10.1016/j.psj.2023.103163 PMC10684806 37980751

[CR17] Joeres, R., Blumenthal, D. B., & Kalinina, O. V. (2026). Addendum: Data splitting against information leakage with DataSAIL. *Nature Communications,**17*, 1597. 10.1038/s41467-025-67495-w PMC12905371 41688426

[CR18] Lawlor, D. A., Harbord, R. M., Sterne, J. A., Timpson, N., & Davey Smith, G. (2008). Mendelian randomization: Using genes as instruments for making causal inferences in epidemiology. *Statistics In Medicine*, *27*, 1133–1163. 10.1002/sim.3034 17886233

[CR19] Lewis, G. D., Asnani, A., & Gerszten, R. E. (2008). Application of metabolomics to cardiovascular biomarker and pathway discovery. *Journal Of The American College Of Cardiology*, *52*, 117–123. 10.1016/j.jacc.2008.03.043 PMC3204897 18598890

[CR20] Liu, J., Wang, X., & Pang, H. (2024). Covariate adjustment in analyzing randomized clinical trials: Approaches, software, and application. In D.-G. Chen (Ed.), *Biostatistics in Biopharmaceutical Research and Development: Clinical Trial Analysis, Volume 2* (pp. 419–447). Springer Nature Switzerland.

[CR21] Meinshausen, N., & Bühlmann, P. (2010). Stability selection. *Journal of the Royal Statistical Society Series B: Statistical Methodology,**72*, 417–473. 10.1111/j.1467-9868.2010.00740.x

[CR22] Moore, L. G., Niermeyer, S., & Zamudio, S. (1998). Human adaptation to high altitude: Regional and life-cycle perspectives. *American Journal of Physical Anthropology*, *107*, 25–64. 10.1002/(SICI)1096-8644(1998)107:27+<25::AID-AJPA3>3.0.CO;2-L 9881522

[CR23] Murray, A. J., Montgomery, H. E., Feelisch, M., Grocott, M. P. W., & Martin, D. S. (2018). Metabolic adjustment to high-altitude hypoxia: From genetic signals to physiological implications. *Biochemical Society Transactions*, *46*, 599–607. 10.1042/bst20170502 29678953

[CR24] Newgard, C. B. (2017). Metabolomics and metabolic diseases: Where do we stand? *Cell Metabolism,**25*, 43–56. 10.1016/j.cmet.2016.09.018 PMC5245686 28094011

[CR25] Simonson, T. S., Yang, Y., Huff, C. D., Yun, H., Qin, G., Witherspoon, D. J., & Ge, R. (2010). Genetic evidence for high-altitude adaptation in Tibet. *Science,**329*(5987), 72–75. 10.1126/science.1189406 20466884

[CR26] Steyerberg, E. W., Vickers, A. J., Cook, N. R., Gerds, T., Gonen, M., Obuchowski, N., & Kattan, M. W. (2010). Assessing the performance of prediction models: A framework for some traditional and novel measures. *Epidemiolog,**21*(1), 128. 10.1097/EDE.0b013e3181c30fb2 PMC3575184 20010215

[CR27] Suhre, K., Shin, S. Y., Petersen, A. K., Mohney, R. P., Meredith, D., Wägele, B., & Gieger, C. (2011). Human metabolic individuality in biomedical and pharmaceutical research. *Nature,**477*(7362), 54–60. 10.1038/nature10354 PMC3832838 21886157

[CR28] Tan, B., O’Dell, D. K., Yu, Y. W., Monn, M. F., Burstein, S., & Walker, J. M. (2010). Identification of endogenous acyl amino acids based on a targeted lipidomics approach. *Journal of Lipid Research,**51*(1), 112–119. 10.1194/jlr.M900198-JLR200 PMC2789771 19584404

[CR29] Tzoulaki, I., Ebbels, T. M., Valdes, A., Elliott, P., & Ioannidis, J. P. (2014). Design and analysis of metabolomics studies in epidemiologic research: A primer on -omic technologies. *American Journal Of Epidemiology*, *180*, 129–139. 10.1093/aje/kwu143 24966222

[CR30] West, J. B. (2012). High-altitude medicine. *American Journal Of Respiratory And Critical Care Medicine*, *186*, 1229–1237. 10.1164/rccm.201207-1323CI 23103737

[CR31] Xia, J., & Wishart, D. S. (2010). MSEA: A web-based tool to identify biologically meaningful patterns in quantitative metabolomic data. *Nucleic Acids Research*, *38*, W71–77. 10.1093/nar/gkq329 PMC2896187 20457745

[CR32] Xia, J., Psychogios, N., Young, N., & Wishart, D. S. (2009). MetaboAnalyst: A web server for metabolomic data analysis and interpretation. *Nucleic Acids Research*, *37*, W652–660. 10.1093/nar/gkp356 PMC2703878 19429898

[CR33] Zucchini, W. (2000). An Introduction to Model Selection. *Journal Of Mathematical Psychology*, *44*, 41–61. 10.1006/jmps.1999.1276 10733857

