# Supplementary material for: Serum metabolic signatures associated with maximal voluntary ventilation in native high-altitude Tibetans
Source: Metabolomics. 2026 Aug 22;22(5):141. doi: 10.1007/s11306-026-02517-2 (PMC13499765; doi:10.1007/s11306-026-02517-2)
Supplement: Supplementary file 1 [file 11306_2026_2517_MOESM5_ESM.docx]

Figure S1. Candidate metabolite numbers across three‑tier hierarchical filtering.

Count of candidate metabolites obtained from univariate regression using covariate‑adjusted MVV residuals. Zero metabolites satisfied FDR < 0.05; 109 metabolites passed P < 0.05 and 255 metabolites passed P < 0.01. Elastic‑net regression returned no non‑zero‑coefficient metabolites for both candidate sets, triggering the predefined fallback strategy selecting top‑five metabolites with smallest univariate P values for further analysis.

Figure S2. Bootstrap resampling (200 iterations) in the training set assessed screening stability. Bars represent inclusion frequencies of the top‑10 metabolites sorted from highest to lowest frequency. Moderate inclusion frequencies indicate weak marginal metabolite‑MVV associations in this high‑altitude Tibetan cohort. Raw results are listed in Supplementary Table S2.
